# Complete Genome Sequence of *Sphingopyxis* sp. Strain PET50, a Potential Polyethylene Terephthalate (PET)-Degrading Bacterium Isolated from Compost

**DOI:** 10.1128/mra.00970-22

**Published:** 2023-01-04

**Authors:** Ana Lago-Maciel, Tue K. Nielsen, Kenneth Jensen, Mette H. Nicolaisen, Rosanna C. Hennessy

**Affiliations:** a Department of Plant and Environmental Sciences, University of Copenhagen, Frederiksberg, Denmark; b Novozymes A/S, Kongens Lyngby, Denmark; University of Southern California

## Abstract

We report the complete genome sequence of a potential polyethylene terephthalate (PET)-degrading bacterium, *Sphingopyxis* sp. strain PET50, isolated from compost.

## ANNOUNCEMENT

An enrichment culture of polyethylene terephthalate (PET)-degrading bacteria was prepared by incubating a piece of plastic debris found in compost at the Sydhavn Genbrugscenter composting facility (Copenhagen, Denmark; 55°38′18.6″N, 12°32′25.5″E) in M9 minimal salts medium, with amorphous polyethylene terephthalate (PET) film as the sole carbon source. An adaptive laboratory evolution (ALE) strategy was applied for 6 months with the same medium treatment to increase the population of efficient plastic degraders. The ALE mixture was spread onto a PET-containing agar plate (1); *Sphingopyxis* sp. strain PET50 was identified by the formation of a degradation clearing zone around the colony and was isolated as a pure culture. Overnight cell cultures (2 mL) were prepared in Reasoner’s 2A (R2A) broth at 150 rpm and 20°C for DNA extraction using the Genomic-tip 20/G kit (10223; Qiagen). The DNA pellet was dissolved in 50 μL of buffer containing 10 mM Tris (pH 8.0) and 50 mM NaCl, and the DNA purity and concentration were determined using NanoDrop and Qubit measurements. A Nanopore sequencing library was prepared using the rapid barcoding kit 96 (SQK-RBK110.96) without DNA size selection. The prepared library was sequenced on the MinION platform with a R9.4.1 flow cell. Default parameters were used for all software unless otherwise specified. Base calling of the raw data was performed using Guppy v. 5.1.12 +0a404b92d (2) with the “super accuracy” model. Adapter sequences were trimmed from the reads using Porechop v. 0.2.4 (3). After trimming, 67,076 reads (151.8 Mb; *N*_50_, 4.2 kbp) were assembled into a single circular contig using Flye v. 2.9-b1774 (4), representing the complete chromosome. The assembly was subsequently corrected with two consecutive rounds of polishing using Medaka v. 1.4.4 (https://github.com/nanoporetech/medaka), with the super accuracy model “r941_min_sup_g507.” The polished genome was annotated using Prokka v. 1.14.6 (5), and taxonomy was predicted using GTDB-Tk v. 2.1.0 (6). The total length of the genome assembly was 3,796,965 bp, while the read depth was 40× and the mean G+C content was 66.71%. Taxonomic classification according to the Genome Taxonomy Database (GTDB) revealed that strain PET50 is a new species of the genus *Sphingopyxis*, with an average nucleotide identity (ANI) below the 95% species threshold.

Genome mining using HMMER v. 3.3.2 (7) revealed the presence of two potential MHETase homologs inside the genome of *Sphingopyxis* sp. strain PET50, identified by using phmmer against the sequence of Ideonella sakaiensis IsMHETase (GenBank protein accession no. A0A0K8P8E7) (8). Additionally, hmmsearch was used together with a publicly available profile hidden Markov model (HMM) of PET-degrading enzymes in the Plastics-Active Enzymes Database (PAZy) (9, 10) to identify putative PET hydrolytic enzymes, albeit unsuccessfully. Thus, *Sphingopyxis* sp. strain PET50 might be a source of novel enzymes involved in the initial steps of the depolymerization of PET oligomers into its constituent monomers.

### Data availability.

This whole-genome shotgun project has been deposited at GenBank under the accession no. CP104207. The version described in this paper is the first version. The raw sequence reads were deposited in the Sequence Read Archive (SRA) under accession no. SRR21734874 and BioSample accession no. SAMN30619208. The Prokka annotations are available from Figshare at https://doi.org/10.6084/m9.figshare.21621384.v1.

## References

[1] Charnock C. 2021. A simple and novel method for the production of polyethylene terephthalate containing agar plates for the growth and detection of bacteria able to hydrolyze this plastic. J Microbiol Methods 185:106222. doi:10.1016/j.mimet.2021.106222. 33865904

[2] Ueno Y, Arita M, Kumagai T, Asai K. 2003. Processing sequence annotation data using the Lua programming language. Genome Inform 14:154–163. doi:10.11234/gi1990.14.154. 15706530

[3] Wick RR, Judd LM, Gorrie CL, Holt KE. 2017. Completing bacterial genome assemblies with multiplex MinION sequencing. Microbial Genomics 3:e000132. doi:10.1099/mgen.0.000132. 29177090 PMC5695209

[4] Kolmogorov M, Yuan J, Lin Y, Pevzner PA. 2019. Assembly of long, error-prone reads using repeat graphs. Nat Biotechnol 37:540–546. doi:10.1038/s41587-019-0072-8. 30936562

[5] Seemann T. 2014. Prokka: rapid prokaryotic genome annotation. Bioinformatics 30:2068–2069. doi:10.1093/bioinformatics/btu153. 24642063

[6] Chaumeil PA, Mussig AJ, Hugenholtz P, Parks DH. 2019. GTDB-Tk: a toolkit to classify genomes with the genome taxonomy database. Bioinformatics 36:1925–1927. doi:10.1093/bioinformatics/btz848. 31730192 PMC7703759

[7] Eddy SR. 2020. HMMER user’s guide: biological sequence analysis using profile hidden Markov models. http://hmmer.org.

[8] Yoshida S, Hiraga K, Takehana T, Taniguchi I, Yamaji H, Maeda Y, Toyohara K, Miyamoto K, Kimura Y, Oda K. 2016. A bacterium that degrades and assimilates poly(ethylene terephthalate). Science 351:1196–1199. doi:10.1126/science.aad6359. 26965627

[9] Buchholz PCF. 2021. Profile hidden Markov model for PETase homologues. DaRUS, Stuttgart, Germany. doi:10.18419/darus-2055.

[10] Buchholz PCF, Feuerriegel G, Zhang H, Perez-Garcia P, Nover L-L, Chow J, Streit WR, Pleiss J. 2022. Plastics degradation by hydrolytic enzymes: the Plastics-Active Enzymes Database—PAZy. Proteins 90:1443–1456. doi:10.1002/prot.26325. 35175626

